# Methylprednisolone improves the quality of liquid preserved boar spermatozoa *in vitro* and reduces polymorphonuclear neutrophil chemotaxis and phagocytosis

**DOI:** 10.3389/fvets.2023.1177873

**Published:** 2023-05-15

**Authors:** Yanbing Li, Hechuan Wang, Shuo Wang, Qun Zhang, Han Zhang, Tianfeng Li, Qian Wang, Minghui Guo, Heze Feng, Yulun Song, Guosheng Wei, Jingchun Li

**Affiliations:** College of Animal Science and Veterinary Medicine, Heilongjiang Bayi Agricultural University, Daqing, Heilongjiang, China

**Keywords:** methylprednisolone, spermatozoa quality, polymorphonuclear neutrophils, chemotaxis, phagocytosis

## Abstract

After artificial insemination, immune cells such as polymorphonuclear neutrophils will be recruited into the genital tract and induce endometrial inflammation, adversely affecting the spermatozoa. This study aimed to analyze the effect of methylprednisolone (MPS) on boar spermatozoa quality of *in vitro* liquid preservation and chemotaxis and phagocytosis of polymorphonuclear neutrophils toward boar spermatozoa. Various concentrations of MPS were added to the extender and analyzed for their effects on spermatozoa motility, kinetic parameters, abnormality rate, total antioxidant capacity (T-AOC) levels, H_2_O_2_ content, mitochondrial membrane potential and acrosome integrity. Testing of MPS on chemotaxis and phagocytosis of polymorphonuclear neutrophils toward spermatozoa induced by lipopolysaccharide (LPS). The results showed that an extender containing 2 × 10^−7^ mol/mL MPS was the most effective for preserving boar spermatozoa during *in vitro* liquid preservation at 17°C. It effectively improved spermatozoa motility, kinetic parameters, T-AOC levels, mitochondrial membrane potential and acrosome integrity, reducing the abnormality rate and H_2_O_2_ content. Meanwhile, the chemotaxis and phagocytosis of polymorphonuclear neutrophils toward spermatozoa under LPS induction were inhibited in a concentration-dependent manner. In conclusion, MPS has positive implications for improving *in vitro* liquid preserved boar spermatozoa quality, inhibiting chemotaxis and phagocytosis of polymorphonuclear neutrophils toward spermatozoa.

## 1. Introduction

In modern swine farms, artificial insemination (AI) is generally used as a fundamental and necessary means of reproduction to maintain normal production conditions (1). After AI, spermatozoa will induce an innate immune response in the uterus after entering the sow’s genital tract, and polymorphonuclear neutrophils (PMNs), as one of the most important cellular components of uterine immunity, will be recruited to the uterus in large numbers 1–2 h after insemination (2–4). As a foreign cell, the spermatozoa are antigenic and the immune cells in the swine’s uterus phagocytosis most of the spermatozoa so that only a small percentage of spermatozoa can reach the fallopian tube and have a chance to unite with the egg (5–7).

In addition to spermatozoa that can induce uterine immunity, bacterial invasion can also cause the onset of the uterine immune response. This is mainly due to mechanical damage and continuous manipulation in AI, which can lead to sterility in the sow and affect the reproductive cycle (8). The bacteria that cause uterine immunity are mainly Gram-negative bacteria such as Enterobacter, Burkholderia, Serratia and Parvimonas (9). Lipopolysaccharide (LPS) released by Gram-negative bacteria affects the endometrial epithelium of the sow. It induces uterine inflammation, so this substance is often used to mimic the inflammatory response *in vitro* (10). This cytotoxicity not only affects sows, but also reduces the quality of boar spermatozoa under *in vitro* liquid preservation. However, substances such as antibiotics have been used to contain the harmful effects of bacteria in the extender (11).

Although the results of boar spermatozoa under *in vitro* liquid preservation have been partially satisfactory, limitations such as short storage time, microbial colonization in extenders and induction of uterine immune response still need improvement (12–14). Therefore, how to improve the quality of *in vitro* liquid preservation of boar spermatozoa and reduce the loss of spermatozoa due to uterine immunity to enhance the fertility rate has become one of the crucial directions of current research on swine AI technology. We have demonstrated in a study that dexamethasone (DEX) improves the quality of *in vitro* liquid preservation boar spermatozoa and inhibits chemotaxis and phagocytosis of PMNs towards spermatozoa (15). Methylprednisolone (MPS), another common glucocorticoid, has a better biological effect compared to DEX, thus completing immunosuppression faster (16). However, few studies have reported the effects of MPS on the quality of boar semen at *in vitro* liquid preservation (17°C) or the chemotaxis and phagocytosis of PMNs toward spermatozoa.

Given this, we believe that adding MPS to extenders may also have a beneficial effect. Glucocorticoids are thought to act directly on cell membrane receptors and modulate the immune system through non-genomic mechanisms to exert anti-inflammatory effects (17). Therefore, our study aimed to assess whether extenders containing MPS are beneficial for *in vitro* liquid preservation of boar spermatozoa by measuring spermatozoa motility, abnormality rate, kinetic parameters, plasma membrane integrity, mitochondrial activity, total antioxidant capacity (T-AOC) activity and H_2_O_2_ content by a computer-assisted spermatozoa analysis (CASA) system or spectrophotometer. The effects of MPS on the chemotaxis and phagocytosis of PMNs induced by LPS were analyzed by blind well chemotaxis chamber test and phagocytosis test.

## 2. Materials and methods

### 2.1. Chemical sources and preparation

Unless otherwise stated, all chemicals used were purchased from Sigma-Aldrich (St. Louis, MO, USA). The modified Modena extender consists of 152.64 mmol/L glucose, 46.64 mmol/L Tris, 26.74 mmol/L sodium citrate, 15.09 mmol/L citric acid, 11.90 mmol/L sodium bicarbonate, 6.98 mmol/mL EDTA-2Na·H_2_O, 1 million U/L streptomycin, 1 million U/L penicillin and 4.00 g/L BSA (prepared just before use). Erythrocyte lysis buffer consists of 150.00 mmol/L NH_4_Cl, 12.00 mmol/L KHCO_3_ and 0.12 mmol/L EDTA. Processed on a sterile bench through a 0.22 μm filter to ensure no bacterial contamination and placed at 4°C. These chemicals were dissolved using pure water and adjusted to the appropriate concentration.

### 2.2. Semen collection and treatment

Six large white boars aged 2–3 years were selected for semen collection from a commercial insemination station (Jingyu Animal Husbandry, Daqing, China). The boars were kept in separate pens with natural light and free access to water and food. Semen was collected once a week for a total of 4 times to obtain the final 24 samples. Samples were stored at 17°C and sent to the laboratory within 2 h. Assessment of spermatozoa viability using CASA (Songjingtianlun Biotechnology, Nanning, China), only if the quality of the spermatozoa meets the requirements (milky white, and slightly smelly, with viability >80%), we will conduct follow-up experiments. Then, the fresh semen was centrifuged at 750 × *g* for 3 min at 17°C (18). The supernatant was then removed, and the spermatozoa pellet was resuspended at a concentration of 1 × 10^8^ cells/mL in modified Modena extender containing different concentrations of MPS (control (0 mol/mL), 1 × 10^−7^ mol/mL, 2 × 10^−7^ mol/mL, 4 × 10^−7^ mol/mL and 6 × 10^−7^ mol/mL). Finally, extended spermatozoa samples were all stored in the incubator, and the temperature was set at 17°C. Spermatozoa quality parameters were detected at each time point (0, 1, 2, 3, 4, and 5) days. Each concentration of MPS samples was set to be repeated four times. Furthermore, fresh semen was used for the chemotaxis and phagocytosis assay of PMNs.

### 2.3. Spermatozoa motility and kinetic parameters evaluation

CASA was used to analyze spermatozoa motility and kinetic parameters. Pipette 10 μL of sample onto a chamber slide (chamber depth 10 μm) with coverslip and preheat for 15 min at 37°C. The standard parameters were set at 30 frames/s. The measured spermatozoa kinetic parameters included average path velocity (VAP), average straight-line velocity (VSL) and average curvilinear velocity (VCL). We defined motility as the percentage of spermatozoa with straightness of path (STR) > 75% and VSL > 25 μm/s. The sample was analyzed using accompanying software, with 5 observation fields randomly selected for each sample and at least 150 spermatozoa recorded per field. This evaluation was technically repeated four times.

### 2.4. Spermatozoa morphology evaluation, T-AOC activity and H_2_O_2_ content determination

Spermatozoa morphology evaluation was assessed by Williams staining as reported by Kavak et al. (19) with appropriate modifications. Briefly, 5 μL aliquots of spermatozoa were placed on a slide and naturally dried. The slides were fixed in absolute ethanol for 2–3 min, naturally dried, then immersed in 0.5% chloramine T for 1–2 min, washed with pure water for 1–2 min, dehydrated quickly in 96% ethanol, naturally dried, then rehydrated with carbonic acid solution for 10–15 min, washed twice, stained with merine, naturally dried. A positive phase-contrast microscope (TS100F; Nikon, Tokyo, Japan) was used for observing the samples at 1000 × magnification (Nikon 20 × 0.40 PLAN objective). Spermatozoa morphology was assessed subjectively by counting 200 spermatozoa, differentiating spermatozoa head deformities and spermatozoa tail deformities (coiled tails, or tails folded at the neck and midpiece). This evaluation was technically repeated four times.

T-AOC activity and hydrogen peroxide (H_2_O_2_) content were determined using the assay kit according to the manufacturer’s operating instructions (Jiancheng Bioengineering Institute, Nanjing, China). Sample preparation was carried out according to the operating instructions. A spectrophotometer (PERSEE, Beijing, China) measured T-AOC activity at 520 nm and H_2_O_2_ content at 405 nm. This evaluation was technically repeated four times.

### 2.5. Mitochondrial membrane potential evaluation

Mitochondrial membrane potential was assessed by JC-1 and propidium iodide (PI) as reported by Ma et al. (20) with appropriate modifications. JC-1 is a fluorescent probe widely used to detect mitochondrial membrane potential. When the mitochondrial membrane potential is at a high level, the probe aggregates in the mitochondrial matrix to form a polymer; when the mitochondrial membrane potential is at a low level, the probe cannot aggregate in the mitochondrial matrix, and the probe is monomeric (21). Briefly, add 100 μL of sample to 400 μL of isotonic buffer diluent containing 1 mmol/L JC-1 and 5 mmol/L PI, mix well and incubate for 30 min at 37°C. Add 15 μL of sample dropwise onto a slide with coverslip, observe by inverted fluorescence microscope (Mshot photoelectric technology, Guangzhou, China) and analyze the sample using the accessory software. Each view was not less than 200 spermatozoa. The heads of spermatozoa with high mitochondrial membrane potential (hMMP) show red fluorescence, while the heads of spermatozoa with medium and low mitochondrial membrane potential show green fluorescence. This evaluation was technically repeated four times.

### 2.6. Spermatozoa acrosome integrity evaluation

Spermatozoa acrosome integrity was assessed by fluorescein peanut agglutinin isothiocyanate (FITC-PNA) as reported by Aboagla et al. (22) with appropriate modifications. Briefly, 30 μL aliquots of spermatozoa were placed on a slide and naturally dried, fixed using methanol for 10 min at 22–25°C. Then 30 μL FITC-PNA solution (100 μg/mL) in phosphate-buffered saline (PBS) was spread over each slide. The slides were incubated in an incubator for 10 min at 37°C, protected from light. The slides were then gently rinsed with PBS and naturally dried. Mounted with 10 μL of an antifade solution to preserve fluorescence. The slide surface is covered with coverslip and sealed with colorless varnish. The acrosome status of the spermatozoa was examined using an epifluorescence microscope (Ti2-U; Nikon, Tokyo, Japan). Each view was not less than 200 spermatozoa. This evaluation was technically repeated four times.

### 2.7. Swine PMNs preparation

Depending on the estrus of the Large White sows, 10–20 mL of peripheral venous blood was collected during the luteal phase using a heparinized vacuum blood collection tube. Delivered to the laboratory within 2 h at 4°C and then processed to collect PMNs. Centrifuge the samples at 1000 × *g* for 10 min at 4°C using a refrigerated centrifuge (SIGMA Laborzentrifugen, Osterode am Harz, Germany), and the buffy coat in the pooled blood plasma from 2 to 4 sows was collected and mixed with 10 mL of PBS in a screw-capped polypropylene centrifuge tube. The buffy coat was washed twice with PBS by centrifugation with 320 × *g* for 10 min at 4°C. The pellet was re-suspended in 4 mL of PBS, carefully layered over 3 mL Histopaque®-1077, and centrifuged at 400 × *g* for 30 min. The supernatant including the mononuclear cells at the interface was removed. Resuspend the particles in 4 mL of erythrocyte lysis buffer. Three minutes later, the solution was centrifuged at 400 × *g* for 10 min, and the lysis procedure was repeated on the subsequent pellet. The PMNs pellet was washed twice at 400 × *g* for 10 min with 5 mL of PBS, resuspended at 1 × 10^8^ cells/mL in 1 mL of PBS, and stored at 4°C until use. The PMNs suspension was used for each replicate within 24 h after preparation. For phagocytosis assay and chemotaxis assay of PMNs, PMNs were also collected from peripheral venous blood of mature boars to ensure that the phagocytotic activities of PMNs are from sows.

#### 2.7.1. PMNs chemotaxis assay

The chemotaxis of PMNs from venous blood to spermatozoa was determined by blind well chemotaxis chamber (BW100; Neuro Probe, Gaithersburg, MD, USA) (23). Briefly, fill the lower chamber with 100 μL of resuspended sample, and place the polycarbonate film (PFA8; Neuro Probe, Gaithersburg, MD, USA) with holes (Ø8 μm) on the upper part of the lower chamber. Connecting tubular assemblies to construct upper chambers and fill with 100 μL of 1 × 10^7^/mL neutrophil TL-HEPES suspension (with different concentrations of MPS). The blind well chemotaxis chamber was incubated at 38.5°C for 90 min, after which the polycarbonate membrane was removed, turned over and placed on a slide and fixed using a clamp. After 15 min of methanol fixation, the polycarbonate membrane was stained with Giemsa solution for 10–15 min. Washing with pure water and treatment with 90% xylene, the polycarbonate membrane was exposed to glacial acetic acid and covered with coverslips. Under a positive phase-contrast microscope with 400× magnification, the number of PMNs passing through the filter was counted at three different areas (0.1520 mm^2^ per area) of each filter. The mean number of PMNs per mm^2^ in three different filter areas was recorded as an observed chemotaxis index of PMNs. This evaluation was technically repeated four times.

#### 2.7.2. PMNs phagocytosis assay

The phagocytosis of PMNs to spermatozoa assay was referred to Matthijs et al. (24) with appropriate modifications. Briefly, 80 μL aliquots of PMNs suspension in TL-HEPES containing various supplements (1 × 10^−6^ g/mL LPS + different concentrations of MPS) were transferred to a polystyrene culture dish. PMNs suspension was mixed with 20 μL spermatozoa suspension and incubated for 60 min at 38.5°C and 5% CO_2_. The final concentrations of PMNs and spermatozoa were 8 × 10^6^ cells/mL and 2 × 10^6^ cells/mL, respectively. After incubation, an equal volume of heparin (40 mg/mL in TL-HEPES) was added to the solution of PMNs and spermatozoa to facilitate the dissociation of agglutinated PMNs. The samples were mixed thoroughly, left for 15 min, and mixed again. Subsamples of 75 μL were fixed by adding 25 μL of 2% (v/v) glutaraldehyde. The fixed samples were mounted on the glass slides and examined under positive phase-contrast microscope at 400× magnification. A minimum of 200 PMNs were counted in each area of the specimen. The percentage of PMNs with phagocytized spermatozoa was recorded as phagocytosis rate. This evaluation was technically repeated four times.

### 2.8. Statistical analysis

All data from each experiment were tested for normality with one-sample Kolmogorov–Smirnov’s test. If some data did not show normality, we arcsine-transformed the variable before the analysis and again checked the normality using one sample Kolmogorove-Smirnov’s test for this parameter. Furthermore, all data from each experiment were checked for the homogeneity of variances using Levene’s test. The test results showed that the replicated data from each experiment were homogeneous. Then, all data were analyzed by one-way Analysis of variance using STATVIEW 5.0 (Abacus Concepts, Berkeley, CA, USA). If the ANOVA *P*-value was less than 0.05, a Bonferroni/Dunn’s HSD test was carried out using the same program. All data were expressed as mean ± SD. Findings were considered significantly different at *P* < 0.05.

## 3. Results

### 3.1. Effects of different concentrations of MPS on boar spermatozoa motility and kinetic parameters

Effects of different concentrations of MPS on boar spermatozoa motility as shown in Table 1. On day 2 of spermatozoa preservation, motility was significantly higher in 1 × 10^−7^ mol/mL MPS, 2 × 10^−7^ mol/mL MPS and 4 × 10^−7^ mol/mL MPS samples than in control samples (*P* < 0.05). On the day 3 of spermatozoa preservation, motility was significantly higher in 1 × 10^−7^ mol/mL MPS and 2 × 10^−7^ mol/mL MPS samples than in 6 × 10^−7^ mol/mL MPS and control samples (*P* < 0.05). On the day 4 of spermatozoa preservation, motility was significantly higher in 1 × 10^−7^ mol/mL MPS, 2 × 10^−7^ mol/mL MPS and 4 × 10^−7^ mol/mL MPS samples than in 6 × 10^−7^ mol/mL MPS and control samples (*P* < 0.05). On the day 5 of spermatozoa preservation, motility was significantly higher in 1 × 10^−7^ mol/mL MPS and 2 × 10^−7^ mol/mL MPS samples than in 6 × 10^−7^ mol/mL MPS and control samples (*P* < 0.05).

**Table 1:** Effects of different concentrations of methylprednisolone (MPS) on boar spermatozoa motility (%).

Treatments	Storage time (day)					
	0	1	2	3	4	5
Control	94.17 ± 1.36^a^	89.49 ± 2.15^a^	75.43 ± 2.13^b^	64.71 ± 2.19^c^	55.57 ± 2.10^c^	46.87 ± 1.74^c^
1 × 10^−7^ mol/mL MPS	94.17 ± 1.36^a^	89.26 ± 2.88^a^	78.71 ± 2.06^a^	70.91 ± 2.06^a^	65.26 ± 2.04^a^	54.56 ± 1.99^a^
2 × 10^−7^ mol/mL MPS	94.17 ± 1.36^a^	90.98 ± 1.17^a^	79.71 ± 1.85^a^	72.68 ± 2.75^a^	67.83 ± 2.68^a^	57.33 ± 1.56^a^
4 × 10^−7^ mol/mL MPS	94.17 ± 1.36^a^	90.46 ± 1.46^a^	79.13 ± 1.49^a^	70.44 ± 2.03^ab^	61.40 ± 2.12^b^	51.15 ± 2.33^b^
6 × 10^−7^ mol/mL MPS	94.17 ± 1.36^a^	90.75 ± 3.55^a^	76.87 ± 1.84^ab^	67.22 ± 2.02^bc^	57.91 ± 2.12^c^	49.18 ± 2.02^bc^

In the same column, values with different letter superscripts mean a significant difference (*P* < 0.05). Similarly here in after.

Effects of different concentrations of MPS on boar spermatozoa VAP as shown in Table 2. On the day 2 and 3 of spermatozoa preservation, VAP was significantly higher in 2 × 10^−7^ mol/mL MPS samples than in control samples (*P* < 0.05). On the day 5 of spermatozoa preservation, VAP was significantly higher in 1 × 10^−7^ mol/mL MPS, 2 × 10^−7^ mol/mL MPS and 4 × 10^−7^ mol/mL MPS samples than in 6 × 10^−7^ mol/mL MPS and control samples (*P* < 0.05), with 2 × 10^−7^ mol/mL MPS samples having the highest VAP.

**Table 2:** Effects of different concentrations of methylprednisolone (MPS) on boar spermatozoa average path velocity (VAP; μm/s).

Treatments	Storage time (day)					
	0	1	2	3	4	5
Control	45.49 ± 0.77^a^	42.72 ± 2.04^a^	34.55 ± 2.25^b^	28.07 ± 1.74^c^	23.16 ± 1.38^c^	18.19 ± 2.54^c^
1 × 10^−7^ mol/mL MPS	45.49 ± 0.77^a^	44.02 ± 0.99^a^	36.10 ± 1.34^ab^	31.90 ± 2.83^ab^	26.77 ± 1.71^b^	23.21 ± 1.52^b^
2 × 10^−7^ mol/mL MPS	45.49 ± 0.77^a^	43.86 ± 2.03^a^	37.90 ± 2.60^a^	33.72 ± 1.78^a^	31.09 ± 2.41^a^	27.48 ± 1.44^a^
4 × 10^−7^ mol/mL MPS	45.49 ± 0.77^a^	43.94 ± 0.97^a^	35.60 ± 1.42^ab^	30.40 ± 3.06^abc^	26.13 ± 1.72^bc^	22.15 ± 1.88^b^
6 × 10^−7^ mol/mL MPS	45.49 ± 0.77^a^	41.36 ± 2.34^a^	34.94 ± 0.88^b^	28.44 ± 4.57^bc^	24.16 ± 2.97^bc^	18.49 ± 1.86^c^

Effects of different concentrations of MPS on boar spermatozoa VSL as shown in Table 3. On the day 3 and 4 of spermatozoa preservation, VSL was significantly higher in 1 × 10^−7^ mol/mL MPS, 2 × 10^−7^ mol/mL MPS and 4 × 10^−7^ mol/mL MPS samples than in 6 × 10^−7^ mol/mL MPS and control samples (*P* < 0.05). On the day 5 of spermatozoa preservation, VSL of each MPS samples was significantly higher than in control sample (*P* < 0.05), with 2 × 10^−7^ mol/mL MPS samples having the highest VSL.

**Table 3:** Effects of different concentrations of methylprednisolone (MPS) on boar spermatozoa average straight-line velocity (VSL; μm/s).

Treatments	Storage time (day)					
	0	1	2	3	4	5
Control	45.19 ± 1.44^a^	38.46 ± 2.34^a^	34.67 ± 1.44^a^	27.70 ± 0.83^c^	19.92 ± 1.75^c^	14.61 ± 1.77^c^
1 × 10^−7^ mol/mL MPS	45.19 ± 1.44^a^	41.37 ± 2.00^a^	36.58 ± 2.80^a^	31.05 ± 0.32^b^	26.64 ± 1.17^b^	20.18 ± 1.32^b^
2 × 10^−7^ mol/mL MPS	45.19 ± 1.44^a^	41.38 ± 4.52^a^	37.40 ± 2.31^a^	33.46 ± 1.59^a^	29.53 ± 2.27^a^	24.76 ± 1.57^a^
4 × 10^−7^ mol/mL MPS	45.19 ± 1.44^a^	40.03 ± 3.34^a^	35.93 ± 2.08^a^	30.25 ± 1.59^b^	26.02 ± 1.76^b^	19.69 ± 2.13^b^
6 × 10^−7^ mol/mL MPS	45.19 ± 1.44^a^	39.04 ± 1.67^a^	35.42 ± 1.83^a^	27.90 ± 1.92^c^	22.33 ± 1.42^c^	17.82 ± 1.79^b^

Effects of different concentrations of MPS on boar spermatozoa VCL as shown in Table 4. On the day 5 of spermatozoa preservation, VCL of each MPS samples was significantly higher than in control sample (*P* < 0.05), with 2 × 10^−7^ mol/mL MPS samples having the highest VCL.

**Table 4:** Effects of different concentrations of methylprednisolone (MPS) on boar spermatozoa average curvilinear velocity (VCL; μm/s).

Treatments	Storage time (day)					
	0	1	2	3	4	5
Control	64.85 ± 1.60^a^	55.27 ± 1.72^a^	44.40 ± 1.66^b^	38.15 ± 1.47^b^	30.43 ± 1.69^c^	23.93 ± 1.06^d^
1 × 10^−7^ mol/mL MPS	64.85 ± 1.60^a^	56.02 ± 2.79^a^	46.10 ± 1.66^ab^	41.64 ± 1.30^ab^	34.52 ± 1.52^ab^	30.74 ± 1.03^b^
2 × 10^−7^ mol/mL MPS	64.85 ± 1.60^a^	57.08 ± 1.99^a^	47.59 ± 1.89^a^	42.97 ± 2.68^a^	36.49 ± 2.07^a^	34.68 ± 2.08^a^
4 × 10^−7^ mol/mL MPS	64.85 ± 1.60^a^	56.89 ± 2.00^a^	45.51 ± 2.10^ab^	39.37 ± 2.54^ab^	32.61 ± 1.17^bc^	29.08 ± 1.43^bc^
6 × 10^−7^ mol/mL MPS	64.85 ± 1.60^a^	56.71 ± 3.03^a^	45.20 ± 1.12^ab^	39.94 ± 2.66^ab^	32.83 ± 2.42^bc^	27.47 ± 1.97^c^

### 3.2. Effects of different concentrations of MPS on boar spermatozoa morphology, T-AOC activity and H_2_O_2_ content

Effects of different concentrations of MPS on boar spermatozoa morphology as shown in Table 5. On the day 4 of spermatozoa preservation, abnormality rate was significantly lower in 1 × 10^−7^ mol/mL MPS, 2 × 10^−7^ mol/mL MPS and 4 × 10^−7^ mol/mL MPS samples than in 6 × 10^−7^ mol/mL MPS and control samples (*P* < 0.05). On the day 5 of spermatozoa preservation, abnormality rate was significantly lower in 1 × 10^−7^ mol/mL MPS, 2 × 10^−7^ mol/mL MPS and 4 × 10^−7^ mol/mL MPS samples than in control samples (*P* < 0.05), with 2 × 10^−7^ mol/mL MPS samples having the lowest abnormality rate. Compared with other MPS samples, the effect of 6 × 10^−7^ mol/mL MPS samples on abnormality rate during the whole storage period shows the opposite result, although the difference is not statistically significant.

**Table 5:** Effects of different concentrations of methylprednisolone (MPS) on boar spermatozoa abnormality rate (%).

Treatments	Storage time (day)					
	0	1	2	3	4	5
Control	4.16 ± 0.46^a^	6.48 ± 0.78^a^	8.48 ± 0.69^a^	10.96 ± 1.82^a^	13.14 ± 1.25^a^	17.48 ± 1.57^a^
1 × 10^−7^ mol/mL MPS	4.16 ± 0.46^a^	5.46 ± 0.69^a^	7.59 ± 0.69^ab^	8.70 ± 1.20^bc^	8.80 ± 0.85^c^	14.46 ± 1.46^b^
2 × 10^−7^ mol/mL MPS	4.16 ± 0.46^a^	5.67 ± 0.69^a^	6.64 ± 0.70^b^	7.74 ± 1.41^c^	8.27 ± 0.59^c^	11.61 ± 2.21^c^
4 × 10^−7^ mol/mL MPS	4.16 ± 0.46^a^	5.96 ± 0.91^a^	7.91 ± 0.54^ab^	9.30 ± 1.00^abc^	10.42 ± 1.73^b^	14.31 ± 1.24^b^
6 × 10^−7^ mol/mL MPS	4.16 ± 0.46^a^	6.41 ± 0.41^a^	8.85 ± 1.53^a^	10.04 ± 1.27^ab^	12.55 ± 0.36^a^	16.75 ± 1.34^ab^

Effects of different concentrations of MPS on boar spermatozoa T-AOC activity and H_2_O_2_ content as shown in Table 6. T-AOC activity was significantly higher in 1 × 10^−6^ g/mL LPS + 2 × 10^−7^ mol/mL MPS samples and 2 × 10^−7^ mol/mL MPS samples than in 1 × 10^−6^ g/mL LPS and control samples at all three test points (*P* < 0.05). T-AOC activity was lower in 1 × 10^−6^ g/mL LPS samples than in control samples, but this difference was only significant at day 3 (*P* < 0.05). The difference in H_2_O_2_ content between 1 × 10^−6^ g/mL LPS + 2 × 10^−7^ mol/mL MPS and 2 × 10^−7^ mol/mL MPS samples was not significant at all three test points (*P* > 0.05). On the day 3 and 5 of spermatozoa preservation, H_2_O_2_ content was significantly lower in 1 × 10^−6^ g/mL LPS + 2 × 10^−7^ mol/mL MPS and 2 × 10^−7^ mol/mL MPS samples than in 1 × 10^−6^ g/mL LPS samples (*P* < 0.05).

**Table 6:** Effects of different concentrations of methylprednisolone (MPS) on boar spermatozoa total antioxidant capacity (T-AOC) activity and H_2_O_2_ content.

Items	Treatments	Storage time (day)		
		1	3	5
T-AOC activity (U/mL)	Control	5.37 ± 1.08^b^	3.55 ± 0.76^b^	1.97 ± 0.50^b^
	1 × 10^−6^ g/mL LPS	4.63 ± 0.42^b^	2.56 ± 0.88^c^	0.99 ± 0.14^b^
	1 × 10^−6^ g/mL LPS + 2 × 10^−7^ mol/mL MPS	6.60 ± 0.68^a^	5.30 ± 0.37^a^	3.95 ± 1.16^a^
	2 × 10^−7^ mol/mL MPS	7.24 ± 0.56^a^	5.61 ± 0.29^a^	3.73 ± 0.91^a^
H_2_O_2_ content (mmol/L)	Control	13.21 ± 1.72^ab^	20.49 ± 2.86^ab^	33.01 ± 1.90^b^
	1 × 10^−6^ g/mL LPS	14.82 ± 1.07^a^	22.91 ± 2.16^a^	36.17 ± 1.43^a^
	1 × 10^−6^ g/mL LPS + 2 × 10^−7^ mol/mL MPS	13.05 ± 1.80^ab^	18.65 ± 2.65^b^	29.03 ± 1.71^c^
	2 × 10^−7^ mol/mL MPS	12.42 ± 2.02^ab^	17.22 ± 2.43^b^	27.74 ± 1.54^c^

### 3.3. Effect of different concentrations of MPS on mitochondrial membrane potential

**Figure 1:**
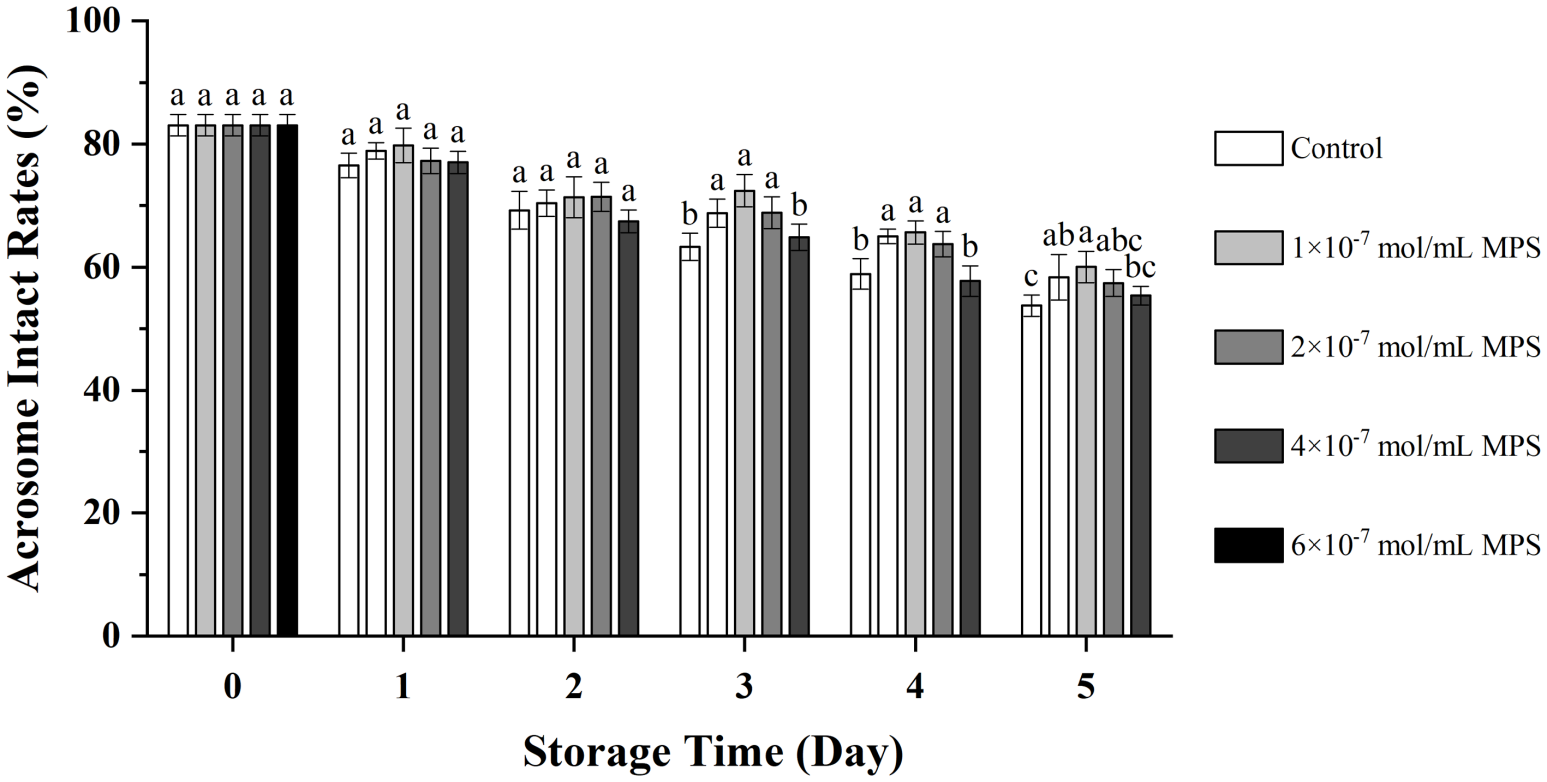
Effects of different concentrations of methylprednisolone (MPS) on mitochondrial membrane potential. In the figure, values with different letters mean a significant difference (*P* < 0.05). Similarly here in after.

Effects of different concentrations of MPS on mitochondrial membrane potential as shown in Figure 1. On the day 3 of spermatozoa preservation, hMMP levels of each MPS samples were significantly higher than in control sample (*P* < 0.05). On day 4 of spermatozoa preservation, hMMP levels was significantly higher in 1 × 10^−7^ mol/mL MPS, 2 × 10^−7^ mol/mL MPS and 4 × 10^−7^ mol/mL MPS samples than in 6 × 10^−7^ mol/mL MPS and control samples (*P* < 0.05). On day 5 of spermatozoa preservation, hMMP levels was significantly higher in 2 × 10^−7^ mol/mL MPS samples than in other MPS samples (*P* < 0.05).

### 3.4. Effect of different concentrations of MPS on acrosome integrity

**Figure 2:**
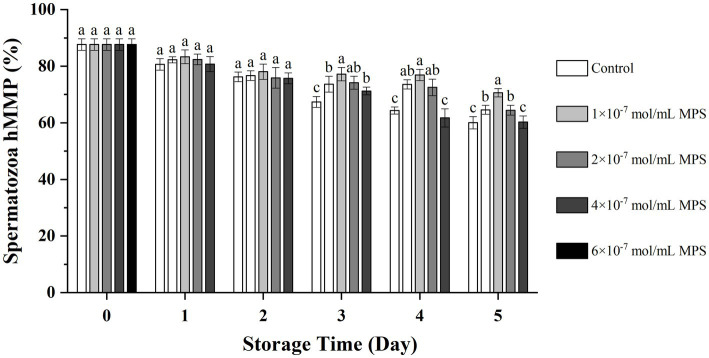
Effect of different concentrations of methylprednisolone (MPS) on acrosome integrity.

Effects of different concentrations of MPS on acrosome integrity as shown in Figure 2. On the day 3 and 4 of spermatozoa preservation, acrosome integrity was significantly higher in 1 × 10^−7^ mol/mL MPS, 2 × 10^−7^ mol/mL MPS and 4 × 10^−7^ mol/mL MPS samples than in 6 × 10^−7^ mol/mL MPS and control samples (*P* < 0.05). On day 5 of spermatozoa preservation, acrosome integrity was significantly higher in 2 × 10^−7^ mol/mL MPS samples than in 6 × 10^−7^ mol/mL MPS and control samples (*P* < 0.05).

### 3.5. Effects of MPS on the chemotaxis of PMNs toward spermatozoa

**Figure 3:**
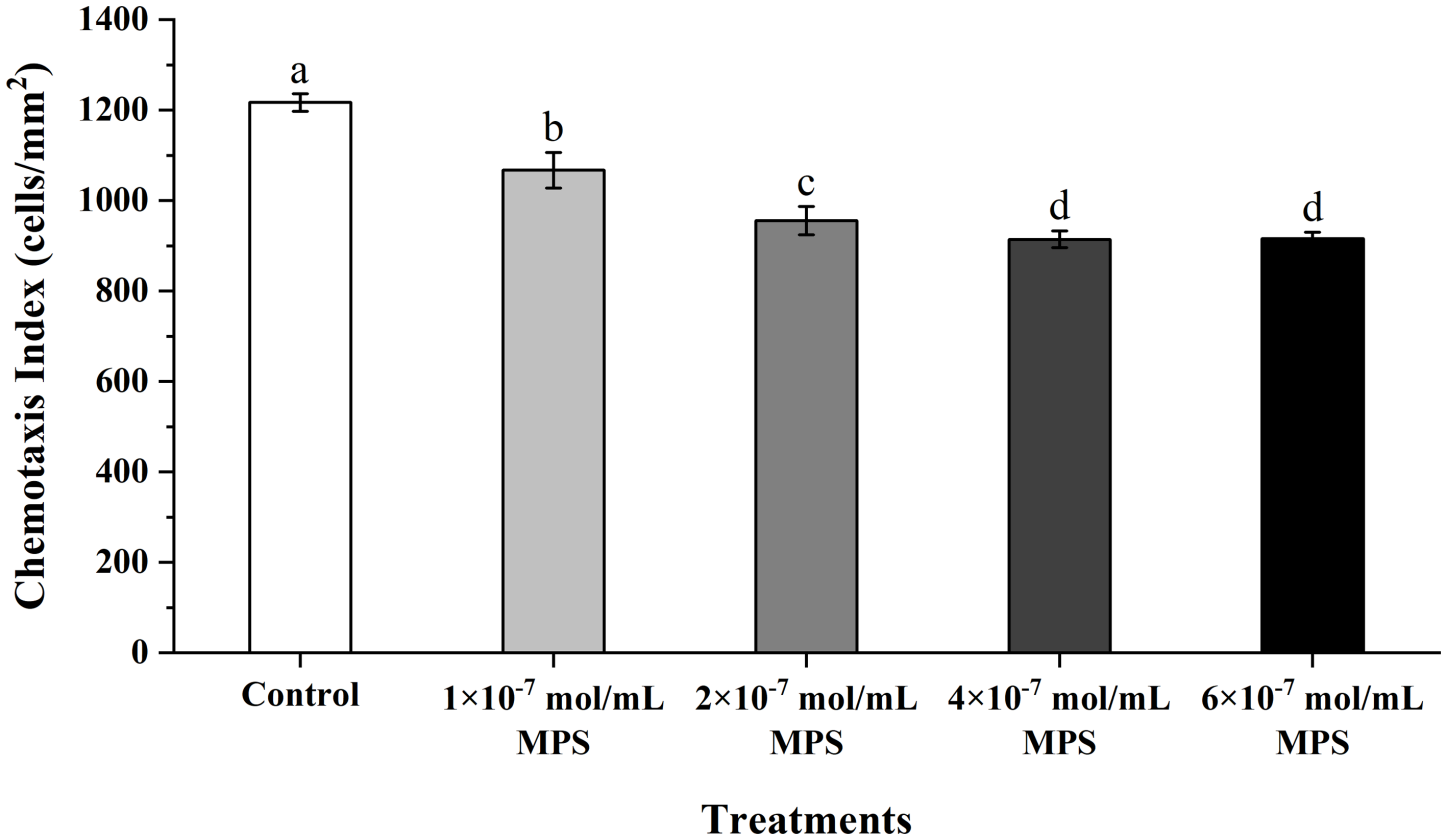
Effects of methylprednisolone (MPS) on the chemotaxis of polymorphonuclear neutrophils (PMNs) toward spermatozoa.

Effects of MPS on the chemotaxis of PMNs toward spermatozoa as shown in Figure 3. The chemotaxis index of PMNs to spermatozoa of each MPS sample is significantly lower than in control sample (*P* < 0.05). And chemotaxis index was reduced in a MPS concentration-dependent manner. In addition, there was no significant difference in the chemotaxis index between the 4 × 10^−7^ mol/mL MPS and 6 × 10^−7^ mol/mL MPS samples (*P* > 0.05).

### 3.6. Effects of MPS on the phagocytosis of PMNs toward spermatozoa

**Figure 4:**
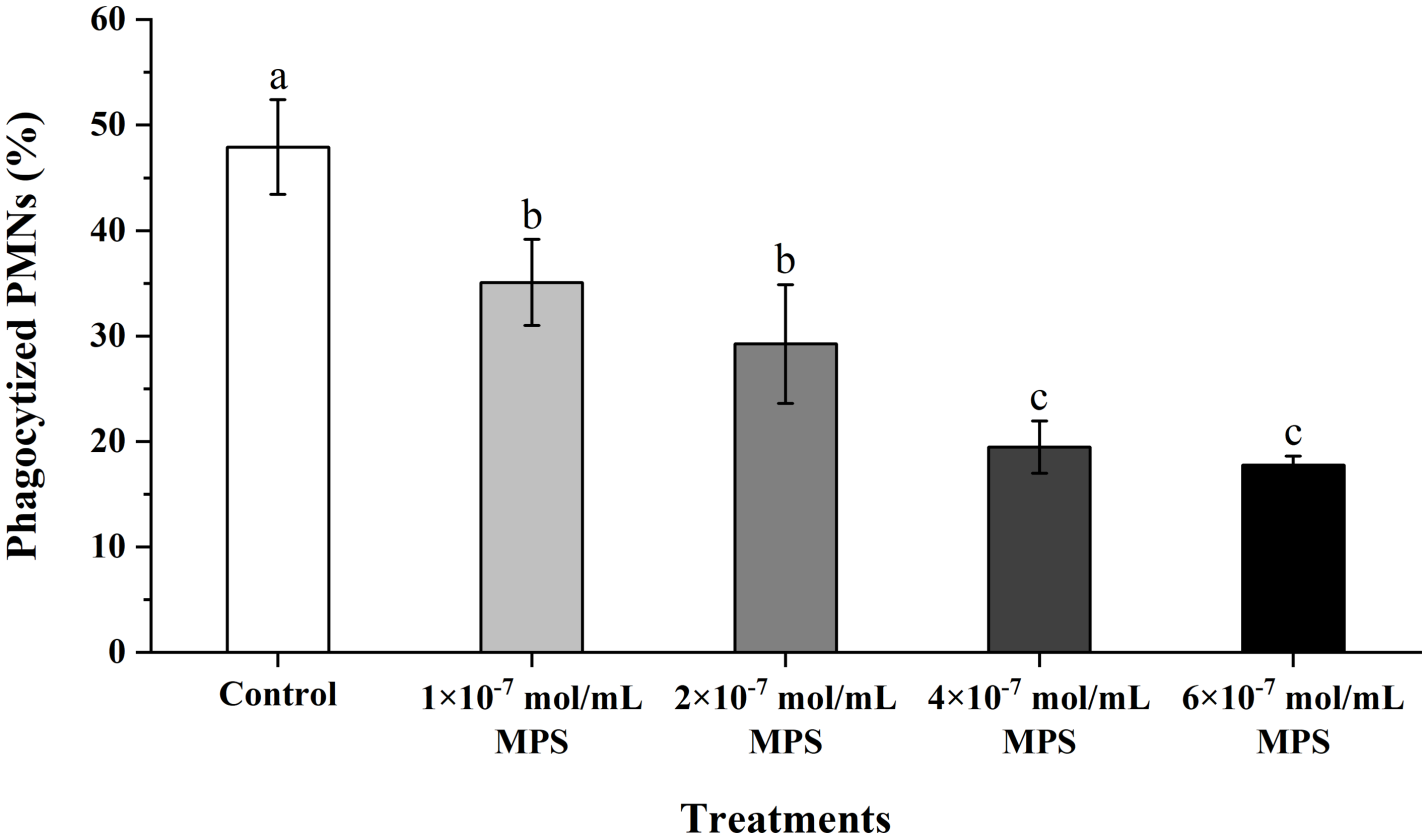
Effects of methylprednisolone (MPS) on the phagocytosis of polymorphonuclear neutrophils (PMNs) toward spermatozoa.

Effects of MPS on the phagocytosis of PMNs toward spermatozoa as shown in Figure 4. The phagocytosis percentage of PMNs to spermatozoa of each MPS sample is significantly lower than in control sample (*P* < 0.05). And phagocytosis percentage was also reduced in a MPS concentration-dependent manner. The difference between the 1 × 10^−7^ mol/mL MPS and 2 × 10^−7^ mol/mL MPS samples was not significant, and the same was true for the 4 × 10^−7^ mol/mL MPS and 6 × 10^−7^ mol/mL MPS samples (*P* > 0.05).

## 4. Discussion

The activity of spermatozoa in the female genital tract is influenced by several factors, the main favorable ones being the increase in temperature and the activation of certain chemicals that increase their motility, thus their ability to reach the site of fertilization more rapidly. However, the boar sperm motility depending on the swine breed, individual differences, reproductive capacity, epidemics and conditions of spermatozoa storage. The higher the spermatozoa motility, the higher the number of spermatozoa reaching the fertilization site and the better the fertilization rate. Our results showed that the spermatozoa motility of the 2 × 10^−7^ mol/mL MPS sample was optimal under 17°C storage conditions, maintaining 57.33% motility at day 5. MPS is based on the addition of double bonds and methyl groups to the molecular structure of prednisone. This further improvement in structure allows MPS to act more swiftly on the cells (25), thus providing faster protection to boar spermatozoa.

The motility pattern of mammalian spermatozoa is generally divided into regular and hyperactivation (26). The hyperactivation of spermatozoa depends mainly on the length of the tail and the thickness of the flagellum (27), so damage to the spermatozoa structure during *in vitro* preservation will directly lead to a reduction in the ability of the spermatozoa to penetrate the egg. Our results show that the spermatozoa kinetic parameters of the 2 × 10^−7^ mol/mL MPS samples were significantly higher than the other samples under *in vitro* liquid storage (17°C).

Spermatozoa morphology is an important predictor of fertilization capacity in semen quality analysis. The morphological abnormalities of spermatozoa can be caused by defects in spermatogenesis and maturation, or by changes in the external environment during spermatozoa preservation. Spermatozoa malformation is generally classified into four types: head defects, neck and mid-section defects, tail defects and excessive cytoplasmic residues (28). Our results showed that at day 5 of spermatozoa preservation, the abnormality rate of 2 × 10^−7^ mol/mL MPS samples was still significantly lower than all other groups, but the high concentration of MPS samples (6 × 10^−7^ mol/mL) instead increased the degree of spermatozoa malformation, which suggested to us the biological toxicity of MPS. Xu et al. (29) showed that MPS protects cell membranes, scavenges free radicals and stabilizes lysosomal membranes.

Total antioxidant capacity activity reflects the total antioxidant capacity of the antioxidants in a system and provides some indication of the overall ability of a system to scavenge reactive oxygen species (ROS). The most common forms of ROS are H_2_O_2_ and O^−^_2_. Under steady-state conditions, superoxide dismutase (SOD) in the mitochondria rapidly converts excess O^−^_2_ to the less reactive H_2_O_2_. H_2_O_2_ is less active but is non-polar and can penetrate the cell membrane environment, directly attacking polyunsaturated fatty acids (PUFA) (30). H_2_O_2_ is a kind of ROS with the most extended half-life, so it is regarded as a beacon of ROS production level (31). Our results show that MPS alleviates the decrease in T-AOC activity and increase in H_2_O_2_ content during *in vitro* liquid preservation under LPS induction.

An early study based on Munich-Wistar rats showed that MPS reduced the level of lipid peroxidation in glomerular tissue and the extent of ROS-mediated kidney injury by enhancing glomerular antioxidant enzyme activity (32). Lee et al. showed that the MPS preserved the rabbit endothelium-dependent vasorelaxation against the attack of ROS in a dose-related manner (33). Although excessive accumulation of ROS can cause irreversible damage to spermatozoa, vital physiological processes such as spermatozoa capacitation and acrosome reaction require the involvement of ROS. Usually, spermatozoa can neutralize ROS through their antioxidant system to avoid oxidative damage. However, in spermatozoa *in vitro* liquid storage, changes in the external environment and temperature will lead to the accelerated production of ROS (34).

Spermatozoa motile by their whiplash tails, and the maintenance of this motility depends on the energy released from ATP hydrolysis (35). A mature spermatozoon typically contains 72–80 mitochondria, the ATP produced is used to maintain intracellular environment stability and provide energy for relevant physiological processes (36, 37). When the ROS produced by the mitochondria increases, the mitochondrial membrane potential will also decrease. Our results showed that the mitochondrial hMMP of boar spermatozoa in the 2 × 10^−7^ mol/mL MPS was higher than other samples under *in vitro* 17°C storage. The mitochondrial hMMP remained at 70.64% at day 5. The mitochondrial membrane potential is closely related to ATP content, and hMMP indicates that the energy metabolism of spermatozoa typically proceeds, whereas this energy metabolism requires oxidative phosphorylation to produce ATP for its maintenance, with a positive correlation (38).

When the spermatozoa enter the oviduct and encounter the egg, the spermatozoa with intact and normal acrosome can generally produce the acrosome reaction and release acrosomal enzymes to cross the zona pellucida into the oocyte and complete fertilization (39). Glucocorticoids can effectively resist free radicals and inhibit calcium influx from stabilizing lysosomes in cells further, thus attenuating the harmful oxidative damage to the cell membrane and more effectively maintaining the complete structure of the cell membrane (40). Therefore, the effect of MPS on acrosome integrity might be similar to that of.

In females during estrus, due to regulating reproductive hormones such as progesterone, many immunocytes migrate into the endometrium to remove foreign cells or substances such as spermatozoa, bacteria and antigens that may subsequently enter the genital tract. After entering the genital tract, spermatozoa will have activated the complement cascade in uterine secretions, after which complement will have induced the chemotaxis with neutrophils and eventually lead to phagocytosis (41). The persistence of this immune response is detrimental to spermatozoa-egg binding after AI, so it is crucial to prevent spermatozoa chemotaxis and phagocytosis by blocking PMNs. Li et al. (3) reported that heparin could inhibit neutrophil chemotaxis and phagocytosis of spermatozoa by binding ligands and blocking the cascade reaction. Alghamdi et al. found by AI of semen with or without seminal plasma (SP) in mares with uterine inflammation, 0.5% (1/22) of mares conceived without SP and 77% (17/22) with SP (42). The beneficial effect of DEX on the inhibition of PMNs chemotaxis and phagocytosis towards spermatozoa has been demonstrated in one of our previous reports (15), while the duration of action of MPS (12–36 h) was shorter compared to dexamethasone (36–54 h) (43), this facilitates faster restoration of the immune barrier function of the sow’s uterus. MPS has been widely reported for its anti-inflammatory role and potent inhibitory effects on immune cells such as PMNs, and it helps regulate the body’s immune system (44). Although chronic or excessive exogenous glucocorticoid exposure is considered a risk to the human fetus, the potential physiologic benefits make short-term or one-time treatment acceptable (45). Our results show that MPS positively affects the inhibition of PMNs chemotaxis and phagocytosis of spermatozoa, which is more substantial with increasing concentrations of MPS.

## 5. Conclusion

In conclusion, our results indicate that MPS enhances the motility and kinetic parameters of boar spermatozoa under *in vitro* liquid preservation (17°C) and inhibits the effects of environmental variations on spermatozoa malformations. Adding MPS to the modified Modena extender reduced radical oxidative damage to spermatozoa, enhanced T-AOC activity and reduced H_2_O_2_ content, which is essential for maintaining boar spermatozoa’s normal physiological functions. Meanwhile, MPS also positively affected spermatozoa hMMP levels and acrosome integrity, essential for spermatozoa insemination ability. In the chemotaxis and phagocytosis assays of PMNs, MPS showed a positive concentration-dependent inhibition of the adverse effects of LPS-induced PMNs on spermatozoa.

## Data availability statement

The original contributions presented in the study are included in the article/supplementary material, further inquiries can be directed to the corresponding authors.

## Ethics statement

The animal study was reviewed and approved by The Animal Experiments Committee of Heilongjiang Bayi Agricultural University. Written informed consent was obtained from the owners for the participation of their animals in this study.

## Author contributions

YL: writing—original draft, conceptualization and methodology. HW: writing—original draft and visualization. SW: writing—review and editing and validation. QZ: writing—review and editing and data curation. HZ: investigation. TL: resources and formal analysis. QW: data curation. MG: investigation and software. HF: resources and investigation. YS: formal analysis. GW: funding acquisition and supervision. JL: project administration and writing—review and editing. All authors contributed to the article and approved the submitted version.

## Funding

This work was supported by Natural Science Foundation of Heilongjiang Province of China (No. LH2022C068) and Heilongjiang Bayi Agricultural University Support Program for San Zong (No. ZRCPY202107).

## Conflict of interest

The authors declare that the research was conducted in the absence of any commercial or financial relationships that could be construed as a potential conflict of interest.

## Publisher’s note

All claims expressed in this article are solely those of the authors and do not necessarily represent those of their affiliated organizations, or those of the publisher, the editors and the reviewers. Any product that may be evaluated in this article, or claim that may be made by its manufacturer, is not guaranteed or endorsed by the publisher.
